# Clinical Studies Examining Intrathecal Drainage for Acute Traumatic Spinal Cord Injury: A Living Systematic Review

**DOI:** 10.1177/2689288X251377024

**Published:** 2025-09-17

**Authors:** Linda Papa, Emily Errante, Ericka Schaeffer, Kimberly Rosenthal, Aiden S. Maciewicz, Nikolay Bugaev, Stephen Shelby Burks, On behalf of the SPINAL TAPPERS Investigators

**Affiliations:** ^1^ Department of Emergency Medicine, Orlando Regional Medical Center, Orlando, Florida, USA.; ^2^ Department of Neurological Surgery, University of Miami Miller School of Medicine, Miami, Florida, USA.; ^3^ Department of Chemistry, University of Central Florida, Orlando, Florida, USA.; ^4^ Department of Surgery, Tufts Medical Center, Tufts University School of Medicine, Boston, Massachusetts, USA.

**Keywords:** cerebrospinal fluid, dural drainage, intrathecal drainage, intraspinal pressure, neurologic outcome, traumatic spinal cord injury

## Abstract

Traumatic spinal cord injury (TSCI) can cause significant and permanent disability. For over 20 years, lumbar cerebrospinal fluid (CSF) drainage has been routinely performed during the surgical repair of thoracoabdominal aortic aneurysms and descending thoracic aortic aneurysms. However, intrathecal CSF drainage has not been adequately evaluated in the setting of acute TSCI. This living systematic review sought to evaluate whether intrathecal catheter CSF drainage to reduce intrathecal pressure (ITP) in the acute postinjury phase was safe and feasible and could improve clinical indices and neurological recovery in patients with acute TSCI. A literature search of PubMed/MEDLINE, Ovid Medline, CINAHL, and Cochrane Database of Systematic Reviews from database inception to March 2024 yielded 1007 potentially relevant articles, 806 were excluded based on title and abstract search and 147 articles underwent full article review. There were two randomized controlled studies, and one cohort study included in the review. Sample sizes ranged from 11 to 22 patients with an age range of 23–67 years. Drains were placed at different times postinjury in each study, with a range of 10–72 h. In the first study, open CSF drainage was used to decrease ITP to 10 mmHg (limit of 10 mL/h). The second study used periodic drainage with up to 10 mL of CSF drained each time (maximum of 30 mL/day). The third study used an open, less restrictive CSF drainage with a target ITP of <10 mmHg. Mean arterial blood pressure and spinal cord perfusion pressure were measured in all three studies. One study evaluated direct intraspinal pressure monitoring. Despite small sample sizes, the three studies demonstrated that intrathecal CSF drainage through a lumbar catheter was feasible and safe acutely after TSCI. Additionally, the results suggest an overall improvement in spinal cord perfusion acutely and trends toward improvements in neurological recovery. There is an important need for much larger prospective trials to evaluate CSF drainage together with other treatment and monitoring strategies to optimize care and improve outcomes. Innovative clinical trial designs could more efficiently evaluate multimodal treatments.

## Introduction

Traumatic spinal cord injury (TSCI) is a debilitating condition with over 768,000 new injuries annually worldwide,^1^ with the most frequent causes of injury being from motor vehicle collisions, falls, gun violence, motorcycle crashes, and diving incidents.^2^ It is a leading cause of paralysis, secondary only to stroke.^3^ Factors that affect recovery include age, baseline health, severity of the initial injury, degree and level of cord injury, and mechanism of the injury, with penetrating injuries incurring worse outcomes than blunt injuries.^4^

Following a primary traumatic injury to the spinal cord, secondary pathophysiological insults can significantly worsen recovery, such as spinal cord edema, vasospasm, ischemia, free radical damage, electrolyte imbalance, excitotoxicity, inflammation, and apoptosis.^5^ These are similar to the secondary insults that follow a traumatic brain injury (TBI). Following TBI, management of increased intracranial pressure (ICP), among other strategies, includes decompressive surgery, and cerebrospinal fluid (CSF) drainage is a standard of care.^6^ Although early decompressive surgery after TSCI is becoming part of standard care,^7^ intrathecal pressure (ITP) monitoring and CSF drainage are not. Spinal cord perfusion pressure (SCPP) is calculated as the difference between mean arterial pressure (MAP) and ITP, therefore, reducing ITP through CSF drainage can increase SCPP and can potentially be used as a therapeutic target for improving outcomes. Maintaining a MAP of 85–90 mmHg for 5–7 days after injury to avoid hypotension is recommended^8^ but this does not address rising intraspinal pressure (ISP) that may impair spinal cord perfusion.

For over 20 years, lumbar CSF drainage has been routinely performed for the surgical repair of thoracoabdominal aortic aneurysms and descending thoracic aortic aneurysms. During these repairs, the spinal cord’s blood supply is vulnerable, and the rate of spinal cord injury (SCI) is between 2% and 8%.^9,10^ A lumbar catheter is introduced into the intrathecal space to allow drainage of CSF to lower ITP and to allow assessment of spinal cord perfusion, with the goal of minimizing spinal cord ischemia and reducing the rate of paraplegia.^10–12^ A survey of European hospitals demonstrated that the majority of centers have formal spinal cord protection protocols that include prophylactic CSF drainage during these aneurysm repair surgeries. While the drainage of CSF to lower ITP and improve SCPP is a common neuroprotective practice in these surgeries, its application in acute TSCI is not well-established.

Given that intrathecal CSF drainage has not been adequately evaluated in the setting of acute TSCI and that practices vary significantly across sites worldwide,^5^ it is important to evaluate the current state of clinical research on the topic. Therefore, this systematic review sought to evaluate the safety and feasibility of intrathecal catheter CSF drainage and whether it could reduce ITP in the acute postinjury phase and improve clinical indices and neurological recovery acutely and long term in patients with acute TSCI.

## Methods

### Literature search

A systematic review was conducted using the Preferred Reporting Items for Systematic Reviews and Meta-Analyses (PRISMA) guidelines.^13^ A literature search of PubMed/MEDLINE, Ovid Medline, CINAHL, and Cochrane Database of Systematic Reviews from database inception to March 2024 was conducted using medical subject headings (MeSH) terms and a keyword search of each database using the Boolean operators OR and AND. The search terms included spinal cord injuries, spinal cord injury, acute spinal cord injury, and TSCI. Search terms for the intervention included spinal decompression, spine decompression, intrathecal decompression, intrathecal hypertension, intrathecal drainage, CSF drainage, as well as expansions of these terms to match synonyms, subterms, or derivatives. For example (((“Spinal Cord Injuries”[Mesh] OR “acute spinal cord injury” OR “traumatic spinal cord injury” OR “spinal cord injury” OR “spinal cord injuries”)) AND ((“spinal decompression”[tiab:∼3] OR “spine decompression”[tiab:∼3] OR “intrathecal decompression”[tiab:∼3] OR “intrathecal hypertension”[tiab:∼3]) OR (“Cerebrospinal Fluid”[Mesh] OR “cerebrospinal fluid”) AND (“Drainage”[Mesh] OR drainage)) AND (English[Filter])). These terms were searched in all fields of publication (e.g., title, abstract, keywords). In addition, the bibliographies and reference lists of all articles and all review articles were evaluated for other potentially relevant articles. The search was limited to English-language articles and studies with human participants.

### Inclusion and exclusion criteria

Studies were considered for inclusion if they met predefined inclusion and exclusion criteria. A table detailing the inclusion and exclusion criteria using PICO (Population, Interventions, Comparators, Outcomes) is presented in Table 1.

**Table 1.** Inclusion and Exclusion Criteria Using PICO Format: Population, Interventions, Comparators, Outcomes

Inclusion	Exclusion
Population	
- Patients of all ages with traumatic spinal cord injury with neurological deficits - Patients with complete or incomplete traumatic SCI at any level	- SCI not due to trauma (e.g., iatrogenic, postsurgical, tumor, hematoma, degenerative disease) - Animal or nonhuman studies
Intervention	
- Use controlled intrathecal cerebrospinal fluid drainage to reduce intraspinal pressure - Drainage through a catheter - Within 7 days of injury	- Durotomy without an intrathecal catheter - Intraspinal pressure monitoring without active drainage
Comparators	
- Controlled intrathecal cerebrospinal fluid drainage versus no drainage	- Comparators not clearly defined
Outcomes	
Efficacy/effectiveness - Neurological outcomes (e.g., American Spinal Injury Association [ASIA] Impairment Scale) - Change in motor scores - Change in sensation Physiological indices Functional or patient reported outcomes Admission/Discharge outcomes - Length of stay - Hospital and ICU admission - Discharge location Safety outcomes - Complications - Adverse events - Death	- Inadequate clinical outcome data
Study designs/publication types	
- Experimental studies (randomized, quasi-experimental) - Observational studies (prospective and retrospective) - Case–control studies - Case series with at least five patients	- Meta-analyses or systematic reviews - Review articles - Opinion papers, editorials, letters - White papers - Books or book chapters - Nonclinical studies - Proceedings/abstracts from meetings - Duplicate publications of the same study (including abstracts)

Neurological outcomes (e.g., Frankel Grade, Spinal Cord Independence measure, change in AIS grade).

Included were studies with: (1) human patients of all ages with SCI secondary to trauma; (2) the use controlled intrathecal CSF drainage to reduce ITP within 7 days of injury; (3) data available on treatment outcomes; and (4) study designs such as experimental studies, observational studies (prospective and retrospective), case–control studies, and case series. Studies were defined as prospective or retrospective according to whether the method of data collection and the endpoints were defined before patient enrollment began.

Studies were excluded if: (1) SCI was not primarily due to trauma; (2) SCI was due to iatrogenic causes; (3) the study did not use an intrathecal catheter for CSF drainage; (4) contained insufficient clinical data; (5) study articles were meta-analyses, review articles, opinion papers, editorials, letters, or books; (6) articles were not available in English; or (7) studies were in nonhuman subjects.

#### Article selection

The titles and abstracts of the publications were screened for relevance; in case of uncertainty regarding the inclusion, the entire text of the article was read. Studies were defined as prospective or retrospective according to whether the method of data collection and endpoints were defined before patient enrollment began. The full texts of the articles were then pooled and reviewed by three different authors to identify articles that met the inclusion criteria. Once the relevant articles were selected, they were reviewed using a standard review form that allowed the reviewers to assess the content of each article in a consistent fashion. A composite evidentiary table was then constructed.

#### Data extraction

Data from included studies were extracted by two authors and confirmed independently by a third author to ensure accuracy. Extraction variables included: authors, year, study design, sample size, age and gender, injury type, inclusion/exclusion criteria, injury mechanism, cord injury level and location, American Spinal Cord Injury Association (ASIA) Impairment Scale at presentation and after treatment, management strategy, details of intrathecal drainage, ITP, treatment complications, and post-treatment functional outcomes. Articles were organized using Covidence® systematic review software (Veritas Health Innovation, Melbourne, Australia).

#### Data synthesis

Due to the heterogeneous nature of data collection and analysis methods, the ability to combine and synthesize results was limited. As such, a descriptive summary was prepared with all included studies constructed into a composite evidentiary table.

## Results

**FIG. 1.**
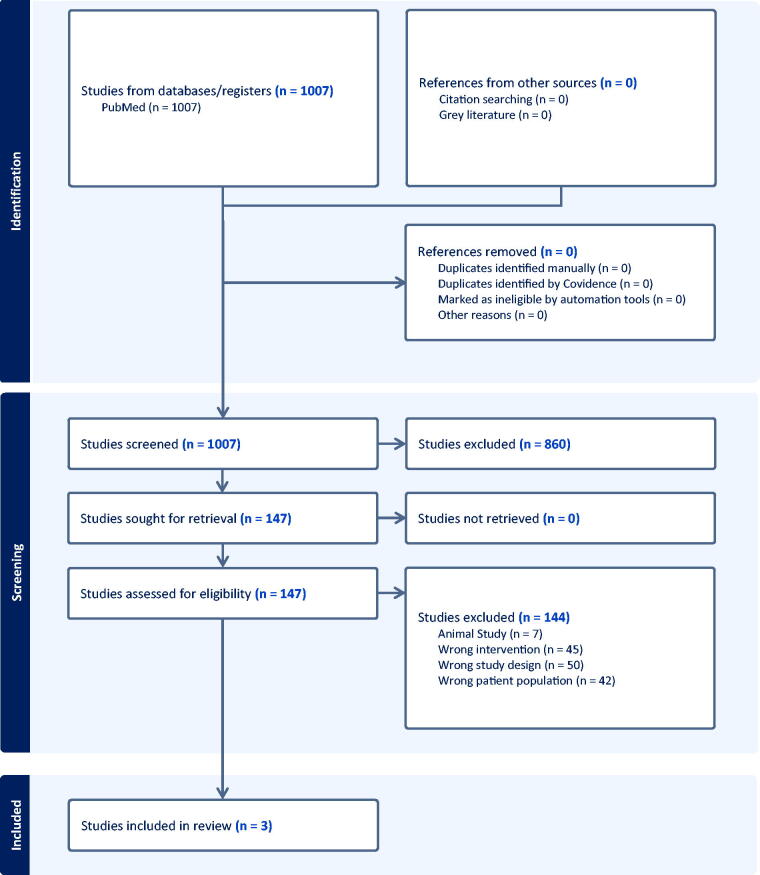
PRISMA diagram for systematic review of clinical studies of dural drainage in spinal cord injury. A literature search of PubMed/MEDLINE, Ovid Medline, CINAHL, and Cochrane Database of Systematic Reviews from database inception to March 2024 yielded 1007 potentially relevant articles, 806 were excluded based on title and abstract search and 147 articles underwent full article review. There were two randomized controlled studies, and one cohort study included in the review. PRISMA, Preferred Reporting Items for Systematic Reviews and Meta-Analyses.

The initial literature search yielded 1007 potentially relevant articles. No duplicates were identified by Covidence or by manual search. Of these, 806 were excluded based on title and abstract search. The remaining 147 articles underwent full article review. After potential conflicts were resolved, three articles met the final inclusion criteria for the systematic review.^14–16^ Reasons for exclusion were animal studies (7), wrong study design (50), wrong patient population (42), and wrong intervention (45). Details of the study selection process are outlined in Figure 1.

There were two randomized controlled studies, and one cohort study included in the review. The evidentiary table summarizing the patient characteristics and methods of each study is in Table 2. ASIA grades A, B, and C were included in all three studies, and total sample sizes ranged from 11 to 22 patients. In all the studies, cervical lesions were included; one study included cervical lesions exclusively, and the two other studies assessed thoracic lesions were also assessed. The age range of patients in the three studies was 23–67 years old, with a mean age of 41–47 years.

**Table 2.** Evidence Table Summarizing Studies Assessing Intrathecal Cerebrospinal Fluid Drainage After Acute Traumatic Spinal Cord Injury

Year/author	Design (e.g., cohort, pre/post, RCT)	Sample size and groups	Ages (years) and sex	Inclusion/exclusion criteria	Level/location of spinal cord injury	ASIA grade	How long after injury when was drain placed	Initial intrathecal pressure (ITP) and subsequent ITP after drainage	How often was fluid drained?	Was MAP measured? Was MAP maximized?
Kwon et al., 2009^14^	RCT	CSF drainage to lower ITP to 10 mmHg (*n* = 11) No CSF drainage (*n* = 11)	Mean 41 years (range 23–66) Male (*n* = 15) Female (*n* = 7)	Inclusion: ASIA grade A-C SCI C3-T11 48 h postinjury undergo neurological examination first-person consent Exclusion: concomitant head, chest, pelvis, other extremities trauma resulting in invasive intervention	C3-T11	A, B, C	Mean 21.6 ± 1.8 h (range 9.5–40 h)	Initial: 13.8 ± 1.3 mmHg Peak ITP (no drainage): 30.6 ± 2.3 mmHg Peak ITP (drainage): 28.1 ± 2.8 mmHg	Free drainage down to a level of 10 mmHg (limit of 10 mL CSF drained/h)	MAP measured, not maintained
Hogg et al., 2020^15^	Cohort	All received CSF drainage (*n* = 13)	Mean 47 years (range 26–67) Male (*n* = 13)	Inclusion: AISA grade A-C 18–70 years surgery performed 72 h postinjury Exclusion: major comorbidities inability to consent penetrating SCI	C3-L1	A, B, C	72 h	N/A	10 mL CSF drained/time, max 30 mL CSF drained/day Average 8.3 times (range 8–14 times)	MAP measured, not maintained
Theodore et al., 2023^16^	RCT	CSF drainage (*n* = 4) no CSF drainage (n = 7)	Mean 41 years in control group (range 27–67) Mean 38 years in experimental group (range 26–48) Male (*n* = 8) Female (*n* = 3)	Inclusion: 18–75 years diagnosis of acute SCI presenting within 24 h postinjury AISA grade A-C C4-C8 neurological level of injury patient completion of neurological exam Exclusion: penetrating SCI significant head injury preexisting neurological or mental disorder prior history of SCI substance and/or psychological disturbances that occurred within the last year patient is prisoner participation in another clinical trial within past 30 days positive pregnancy test immune system disorders cancer	C4-C7	A, B, C	Mean 15.2 ± 7.0 h (CSF drainage group) Mean 9.87 ± 4.1 h (no CSF drainage group)	Initial: N/A Final: 5.3 ± 2.5 mmHg (CSF drainage group); 15 ± 3.0 mmHg (no CSF drainage group)	Free drainage down to a target ITP of <10 mmHg for 5 days Recorded hourly	MAP measured Elevated to 85–90 mmHg in no CSF drainage group for 5 days Beginning during surgery, elevated to 100–110 mmHg in CSF drainage group

CSF, cerebrospinal fluid; RCT, randomized controlled trial; SCI, spinal cord injury.

Drains were placed at different times postinjury in each study, with a range of 10–72 h. In the study by Kwon et al., a free or open CSF drainage method (except that it was closed when the patient could not be examined neurologically) was used to decrease ITP to 10 mmHg with a limit of 10 mL/h. In the cohort study by Hogg et al., a periodic drainage method was used with up to 10 mL of CSF drained each time with a maximum of 30 mL/day. A less restrictive CSF drainage method was used in the study by Theodore et al., with a target ITP of <10 mmHg. MAP and SCPP were measured in all three studies. Table 3 summarizes the outcomes and complications in the included studies.

**Table 3.** Evidence Table Summarizing Outcomes and Complications in Studies Assessing Intrathecal Cerebrospinal Fluid Drainage after Acute Traumatic Spinal Cord Injury

Year/author	Primary and secondary outcome measures	Summary of results	Improvement in ASIA grade	Complications
Kwon et al., 2009^14^	Changes in ITP with surgical decompression Changes in ITP postoperatively Volume of drained CSF Spinal cord perfusion pressure during postoperative monitoring CSF pressure waveform Motor recovery	After spinal cord was decompressed, ITP in all but one patient increased During postoperative period, ITP in patients in both groups generally decreased; periods where ITP increased beyond preoperative peak Volume of CSF drained during postoperative period in CSF drainage group was 117.8 ± 42.8 mL SCPP in postoperative period was trending towards significance between the two groups Significant relationship between MAP and SCPP When catheters were inserted, CSF waveform was flat with no pulsations; after decompression, waveform changed dramatically with an increase in pressure No significant differences in motor score change at 6 months between two groups	No significant differences between groups when comparing segmental motor recovery of patients with cervical injuries only Study not properly powered to assess this directly Similar motor scores suggest no detrimental or adverse effect of CSF drainage on neurological outcome	One patient in no-drainage group complained of transient headache while intrathecal drain was in place One patient in no-drainage group suffered posterior cervical wound infection and developed meningitis; infections occurred over 1 week after catheter had been removed from spine; successfully treated with antibiotics
Hogg et al., 2020^15^	ISP versus CSFP waveform shapes ISP versus CSFP signals SCPP, sPRx, SCPP_opt_ and metabolites Effect on ISP of CSF drainage Cord swelling and CSFP versus ISP correlation	ISP waveforms comparable to intracranial pressure waveform with three major peaks; CSFP waveforms were flatter than ISP waveforms and less pulsatile When averaged over entire monitoring period, ISP was higher than CSFP by ∼5 mmHg No correlation between SCPP_(csf)_ versus SCPP_(ISP), _sPRx_(csf)_ versus sPRx_(ISP)_, or SCPP_opt(csf)_ versus SCPP_opt(ISP)_ In 12/13 patients, drained ∼10 mL CSF through lumbar catheter on several occasions; CSF drainage had variable effect on ISP Patients with less swollen spinal cords had stronger positive correlations between CSFP and ISP signals	At follow-up, 54% of patients had improved by one or more AIS grade 38% remained the same at follow-up One patient (8%) deteriorated by one grade at follow-up	Lumbar drain stopped working in three patients; was resited or removed 1 patient with preexisting and poorly controlled diabetes developed postoperative septicemia followed by wound infection 17 days after surgery; infection treated successfully with IV antibiotics and wound washout CSF leak from the probe skin exit was noted in 4/13 patients; successfully stopped
Theodore et al., 2023^16^	Mean ITP and spinal cord perfusion pressure over 120 h after CSF drainage initiation Change in ASIA grade between baseline and 6-month follow-up Motor and sensory score changes Adverse events Voluntary contraction and sensation of the anal sphincter	CSF drainage patients had a mean MAP 105.4 mmHg; no CSF drainage patients had a mean MAP of 92.1 Mean ITP in the CSF drainage patients was 5.3 mmHg; no CSF drainage patients had a mean ITP of 15 Combined CSF drainage and MAP augmentation produced a mean spinal cord perfusion pressure of 101 mmHg in the CSF drainage group; significantly higher than the no CSF drainage group (77 mmHg) Motor and sensory function improved in both groups	ASIA grade improvements were similar between groups Of the seven patients were completed the 180-day follow-up, three improved one grade and one improved two grades in the no CSF drainage group while two improved one grade and one improved two grades in the CSF drainage group	Respiratory failure in three patients Stroke in one patient Seizure in one patient Decubitus ulcer (sacral) in one patient Deep venous thrombosis in three patients Autonomic dysreflexia in one patient No cases were found to be related to the study procedures Adverse events were similar between groups No patients experienced CSF leak

ASIA, American Spinal Injury Association Impairment scale; ITP, intrathecal pressure; MAP, mean arterial blood pressure; SCPP, spinal cord perfusion pressure (MAP − ITP); ISP, intraspinal pressure; CSF, cerebrospinal fluid; RCT, randomized controlled trial; SCI, spinal cord injury; CSFP, cerebrospinal fluid pressure.

The randomized control trial by Kwon et al.^14^ sought to evaluate the role of CSF drainage and to measure ITP in the acute phase of SCI. The preoperative and intraoperative maximum ITPs in all patients were 13.8 and 21.7 mmHg, respectively. Postoperatively, after decompressive surgery, the average maximum ITP increased in both the CSF drainage (*n* = 11) and no-drainage (*n* = 11) groups to peaks of 28.1 mmHg in the CSF drainage group and 30.6 mmHg in the no-drainage group. These increases were only significant in the no-drainage group. Although mean postoperative SCPP was higher in the CSF drainage group (65.5 mmHg) than in the no-drainage group (58.7 mmHg), MAP was found to correlate significantly with SCPP, and the difference was not statistically significant. During the first 36–40 h postoperatively, the ITPs were similar between the drainage and no-drainage groups, but after 40 h, the ITP was lower in the patients undergoing CSF drainage. During the first 48 h in the postoperative period, a number of patients lost their pulsatile CSF pressure waveform and reverted to the flat pattern, reflective of a blockage of CSF flow across the injury site (confirmed on MRI).

Neurological deterioration and signs of CSF leakage did not occur in either group while the intrathecal catheter was in place, and there were no major adverse events specifically linked to catheter insertion or drainage. Neither group exhibited infection of either the surgical site or central nervous system due to catheter placement and/or drainage; nor did either group develop symptoms such as nausea/vomiting. None of the 11 patients randomized to the CSF drainage group complained of a headache. Long-term neurological differences could not be definitively explored due to the small sample size, but there were no significant differences in the motor score change at 6 months between the two groups (21.0 vs. 15.4 motor points, respectively).

In the 2020 study by Hogg et al.,^15^ 13 patients with acute TSCI enrolled into the ISCoPE trial^17^ were monitored simultaneously from the injury site (requiring surgery to expose the dura and insert probes on the surface of the injured cord under a microscope) and from the lumbar intrathecal space to define the relationship between ITP versus ISP, as well as SCPP_(ITP)_ versus SCPP_(ISP)_, and determined the effect of draining lumbar CSF (intrathecal) on ISP. ISP was generally higher than ITP by about 5 mmHg, but the correlation between ITP and ISP was variable, with periods of positive correlation and periods of no correlation. CSF drainage had a variable effect on ISP: 58% had no change in ISP, 33% had a significantly small (<5 mmHg) reduction, and 8% had a significant large (5–10 mmHg) reduction. Patients with less swollen cords on MRI had a stronger positive correlation between their ITP and ISP. Conversely, when the injured cord was swollen and compressed against the dura (thus no CSF around the injury site), the correlation was poor. CSF drainage did not reliably reduce ISP in most patients, and this was possibly due to the lack of CSF around the swollen injury site. At 6-month follow-up, most patients (54%) had improved by one or more AIS grades, some (38%) remained the same, and one patient (8%) deteriorated by one AIS grade.

In three patients (23%), the lumbar drain stopped working and had to be replaced at another site or removed. CSF leak from the probe skin exit site was observed in 4/13 (31%) patients and was successfully stopped with suturing. One diabetic patient developed urosepsis followed by a wound infection at 17 days and was successfully treated with washout and antibiotics. Finally, 6/13 patients (46%) had asymptomatic pseudo-meningoceles on MRI at 2–3 months that resolved spontaneously after a year.

The randomized control trial by Theodore et al. in 2023 evaluated the safety and feasibility of CSF drainage to improve SCPP and outcomes after acute TSCI. The control group (*n* = 7) received MAP elevation only, and the intervention group (*n* = 4) received MAP elevation and CSF drainage to achieve an ITP <10 mmHg. The patients in the CSF drainage group had significantly lower mean ITP (5.3 mmHg) than the control group (15 mmHg). MAP measurements in the control and CSF drainage arms were 105 and 92 mmHg, respectively. SCPP was significantly higher in the CSF drainage group (101 mmHg) compared to the control group (77 mmHg). ASIA grade improvements were similar between groups. Although light sensation and pinprick scores did not significantly differ between groups, total motor scores improved by 15 and 57 points in the control and CSF drainage groups, respectively, at 6 months. Adverse events were similar between cohorts, and no patient experienced CSF leak.

## Discussion

This systematic review sought to examine the feasibility, safety, and clinical use of intrathecal CSF drainage via lumbar catheter to reduce ITP in the acute postinjury phase of TSCI. The three included studies provided important insights into CSF drainage relative to ITP, ISP, and SCPP monitoring acutely after TSCI. Although there were trends in improved ITP, intrathecal drainage of lumbar CSF did not significantly improve cord perfusion or neurological function in most patients in the studies by Kwon and Hogg. However, there were significant decreases in ITP and increases in SCPP in the study by Theodore, along with improvements in total motor scores. One possible explanation is that the CSF drainage protocol in the study by Theodore was considerably less conservative and purposefully targeted drainage to an ITP of <10 mmHg, whereas the studies by Kwon and Hogg used much stricter drainage parameters.

In addition to CSF drainage and ITP monitoring, the study by Hogg also included direct ISP monitoring, a relatively novel technique for monitoring ISP and SCPP in patients with SCI that requires surgery to insert probes intradurally at the site of injury under a microscope.^18^ It is more invasive than intrathecal catheter placement and is analogous to monitoring cerebral perfusion pressure in patients with TBI. The correlation between ITP and ISP was variable, with periods of positive correlation and periods of no correlation that appeared to be related to intermittent cord compression. CSF drainage was not effective when the injured cord was swollen and compressed against the dura. The dura surrounding the injury site is an important cause of spinal cord compression after TSCI.^17^ Moreover, intrathecal CSF pulsations were also influenced by cord compression around the injury site. Based on MRI findings, the presence of these waveforms appeared indicative of the patency of the intrathecal space from the cranium to the lower lumbar spinal canal. Conversely, the absence of pulsations indicated a blockage of the intrathecal space, likely from the injury site. Given that several patients in these studies exhibited alternating periods of pulsations and no pulsations suggest that cord compression is a dynamic process, even in the postdecompression phase of recovery.^15^ Therefore, ITP does not appear to be representative of the ISP when the subdural space is occluded by the swollen spinal cord.

Duraplasty of the thecal sac at the level of injury is another procedure to potentially counter increased ITP.^19^ Durotomy and duraplasty following decompressive surgery in TSCI has been likened to durotomy following decompressive craniectomy for TBI by helping to more thoroughly decompress the spinal cord and improve CSF circulation and spinal cord perfusion.^17^ Durotomy and duraplasty were not performed in any studies reviewed and are still not part of the standard of care in TSCI.

MAP was measured in all three studies and followed the recommended targets of 85–90 mmHg. Since SCPP is determined by the difference between MAP and ISP, any increase in ISP will subsequently reduce SCPP, regardless of MAP targets. Given that SCPP is influenced by many factors, it is likely that a multimodal approach to TSCI management will be required to optimize outcomes.

Although this review focuses on ITP and lumbar drainage as a means of improving spinal cord perfusion after TSCI, it should not be considered in isolation. There are several physiological parameters, such as MAP, ISP, and SCPP, that have significant effects on spinal cord perfusion. There is a need to define optimal parameters, such as ITP, ISP, MAP, and SCPP, to guide the management of TSCI using strategies learned from trials in TBI. For instance, TBI management includes optimizing ICP and cerebral perfusion pressure to prevent secondary brain damage. Similar approaches will be necessary for TSCI.

Dural drainage in these studies appeared to be feasible and safe. The two randomized trials did not demonstrate any major adverse events from intrathecal drainage. Although the complications directly related to dural drainage were not major and were successfully treated in the study by Hogg, they were relatively more frequent. Potential complications from intrathecal catheter placement for ITP monitoring and particularly for “injury site” ISP monitoring can include mechanical spinal cord damage, hematoma, dural CSF leak (with or without pseudomeningocele), leaks through the skin, wound breakdown, local and CSF infections such as meningitis, and mechanical probe issues such as displacement, breakage, or retention requiring surgical removal.^17^

There is a fundamental need for a uniform research methodology to be applied to future TSCI research. Standardizing how and when data is collected relative to the time of injury, using standard data collection elements, and applying consistent performance and outcome measures will enable data aggregation and meta-analyses of different studies and data sets. This is particularly important given the low incidence rate of TSCI.

## Conclusion

The three studies demonstrate that intrathecal CSF drainage through a lumbar catheter is feasible and safe acutely after TSCI. Additionally, the results suggest an overall improvement in spinal cord perfusion acutely and trends toward improvements in neurological recovery. However, given the small sample sizes, results must be interpreted with caution. There is a critical need for more prospective trials to systematically evaluate CSF drainage together with other treatment strategies and monitoring techniques to optimize physiological parameters and maximize spinal cord perfusion to improve neurological outcomes. Much larger cohorts of patients using innovative clinical trial designs to efficiently evaluate multimodal treatments could provide more definitive answers to optimize care.

## Transparency, Rigor, and Reproducibility Statement

This systematic review demonstrates transparency, scientific rigor, and reproducibility through the following aspects. This review was performed following recommendations of the PRISMA. Four databases were searched for this systematic review: PubMed/MEDLINE, Ovid Medline, CINAHL, and Cochrane on March 30,2024. Reference lists of the articles were also reviewed to ensure all possible articles meeting criteria were captured. Following the search, all references were uploaded to Covidence (Covidence systematic review software, Veritas Health Innovation, Melbourne, Australia). The initial literature search yielded 1007 potentially relevant articles. No duplicates were identified by Covidence or by manual search. Of these, 806 were excluded based on title and abstract search. The remaining 147 articles underwent full article review. They were reviewed using a standard review form that allowed the reviewers to assess the content of each article consistently. Data from included studies were extracted by two authors and confirmed independently by a third author to ensure accuracy. After any discrepancies were resolved, three articles met the final inclusion criteria for systematic review. The authors agree to provide the full content of the article upon request.

## Abbreviations Used

ASI: American Spinal Cord Injury Association impairment scale
ASIA: American Spinal Cord Injury Association
CSF: Cerebrospinal fluid
ICP: Intracranial pressure
ICU: Intensive Care Unit
ISP: Intraspinal pressure
ITP: Intrathecal pressure
MAP: Mean arterial pressure
SCI: Spinal cord injury
SCPP: Spinal cord perfusion pressure
TBI: traumatic brain injury
TSCI: traumatic spinal cord injury
